# Recent Findings Concerning PAMAM Dendrimer Conjugates with Cyclodextrins as Carriers of DNA and RNA

**DOI:** 10.3390/s90806346

**Published:** 2009-08-17

**Authors:** Hidetoshi Arima, Keiichi Motoyama

**Affiliations:** Graduate School of Pharmaceutical Sciences, Kumamoto University, 5-1 Oe-honmachi, Kumamoto 862-0973, Japan; E-Mail: motoyama@kumamoto-u.ac.jp (K.M.)

**Keywords:** cyclodextrin, PAMAM dendrimer, carrier, DNA, siRNA, shRNA

## Abstract

We have evaluated the potential use of various polyamidoamine (PAMAM) dendrimer [dendrimer, generation (G) 2-4] conjugates with cyclodextrins (CyDs) as novel DNA and RNA carriers. Among the various dendrimer conjugates with CyDs, the dendrimer (G3) conjugate with α-CyD having an average degree of substitution (DS) of 2.4 [α-CDE (G3, DS2)] displayed remarkable properties as DNA, shRNA and siRNA delivery carriers through the sensor function of α-CDEs toward nucleic acid drugs, cell surface and endosomal membranes. In an attempt to develop cell-specific gene transfer carriers, we prepared sugar-appended α-CDEs. Of the various sugar-appended α-CDEs prepared, galactose- or mannose-appended α-CDEs provided superior gene transfer activity to α-CDE in various cells, but not cell-specific gene delivery ability. However, lactose-appended α-CDE [Lac-α-CDE (G2)] was found to possess asialoglycoprotein receptor (AgpR)-mediated hepatocyte-selective gene transfer activity, both *in vitro* and *in vivo*. Most recently, we prepared folate-poly(ethylene glycol)-appended α-CDE [Fol-PαC (G3)] and revealed that Fol-PαC (G3) imparted folate receptor (FR)-mediated cancer cell-selective gene transfer activity. Consequently, α-CDEs bearing integrated, multifunctional molecules may possess the potential to be novel carriers for DNA, shRNA and siRNA.

## 1. Introduction

Gene therapy is emerging as a potential strategy for the treatment of genetic diseases, cancers, cardiovascular diseases and infectious diseases [1]. Clinical trials employing over 1,500 gene therapy protocols have been carried out for various diseases [2–4]. Recently, gene silencing induced by small interfering RNA (siRNA), RNA interference (RNAi), became a powerful tool of gene analysis and gene therapy [5–7]. Likewise, vector-based short-hairpin RNAs (shRNA) expression systems have been developed in order to prolong the RNAi effect [8]. However, standard therapeutic use of DNA (gene) and siRNA in clinical settings in humans has been hampered by the lack of effective methods to deliver these nucleic acid drugs into the diseased organs and cells [9–12]. For these reasons, the improvement in transfer activity of a non-viral vector (carrier) is of utmost importance [13–15].

The two gene delivery methods are well known: the viral method and the non-viral method [16,17]. In general, viral vectors have, however, safety risks such as immunogenicity, oncogenicity and potential viral recombination to be solved [18–21]. Hence, more attention is given to the applications of non-viral vectors, because the non-viral method has the profound advantage of being non-pathogenic and non-immunogenic. The non-viral method is further subdivided into two methods, i.e. physical delivery and chemical delivery methods, and the latter method includes three types: lipofection, polyfection and lipopolyfection methods [22,23]. Recently, numerous polycations and polymer micelle have been used for formulating gene, shRNA and siRNA into complexes now termed “polyplexes”. Polycations include histons, polylysine, cationic oligopeptides, polyethyleneimine (PEI), polypropyleneimine (PPI), dendrimers, poly(2-(dimethylamino)ethyl methacrylate and chitosan [13]. The potential use of polyelectrolyte complex micelles for delivering nucleic acid drugs has also been reported [23–27].

## 2. Polyamidoamine (PAMAM) Starburst™ Dendrimers (Dendrimers) as DNA, shRNA and siRNA Carriers

**Figure 1.**
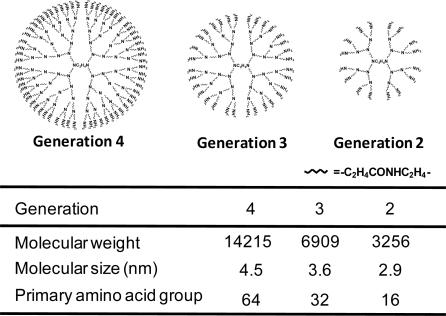
Chemical structures of PAMAM Dendrimers (G4, G3, G2).

Dendrimers, which were developed by Tomalia *et al*., are biocompatible, non-immunogenic and water-soluble, and possess terminal modifiable amine functional groups as the sensor for binding various targeting or guest molecules [28,29]. Unlike classical polymers, dendrimers have a high degree of molecular uniformity, narrow molecular weight distribution, specific size and shape characteristics, and a highly-functionalized terminal surface [30]. The family of cationic dendrimers with low generations is shown in Figure 1. Dendrimers can form complexes with nucleic acid drugs such as plasmid DNA (pDNA), shRNA and siRNA through electrostatic interactions and bind to glycosaminoglycans (heparan sulfate, hyaluronic acid and chondroitin sulfate) on cell surface [31,32], and have been shown to be more efficient and safer than either cationic liposomes or other cationic polymers for *in vitro* gene transfer [33,34]. In addition, the high transfection efficiency of dendrimers can not only be due to their well-defined shape but also be caused by the low pKa of the amines (3.9 and 6.9). The low pKa allows the dendrimer to buffer the pH changes in the endosomal compartment [35], i.e., the enhanced transfection has been attributed to the dendrimer acting as a proton sponge, similar to polyethyleneimine (PEI) in the acidic endosomes, leading to osmotic swelling and lysis of endosomes/lysosomes [36]. It is evident that the nature of dendrimers as non-viral vectors depends significantly on their generation (G). Gene transfer activity of dendrimers with high generations is likely to be superior to that of low generation [32,37]. Furthermore, maximal transfection efficiency using dendrimer (G6) was reported to be obtained, compared to higher generation’s dendrimers, possibly due to rigid structure and cytotoxicity of the dendrimers with higher (>G7) generation [38]. In fact, the cytotoxicity of dendrimers augmented as the generation increased.

Therefore, there has been a growing interest in developing low generation dendrimers (<G4) because of their extremely low cytotoxicity [39]. It should be noted that PAMAM dendrimers developed by Szoka *et al*. represents a new class of transfection regents based on activated-dendrimer technology, removing some of the branches [40]. Indeed, Superfect™ has been reported with enhanced transfection activities due to the increased flexibility of the fractured dendrimers that enable them to be compact when forming complexes with DNA and to swell when released from DNA [41].

Surprisingly, anti-inflammatory effects and apoptotic activity of dendrimers were recently reported, although the detailed mechanisms are still unknown [42,43]. Hereafter, these pharmacological and physiological properties of dendrimers should be considered, and some improvement of the unexpected activity of dendrimers through conjugation with functional moieties may be required.

## 3. α-Cyclodextrin Conjugates with Dendrimers (α-CDE) as DNA, shRNA and siRNA Carriers

Cyclodextrins (CyDs) were first isolated approximately 100 years ago and were characterized as cyclic oligosaccharides [44–46]. The α-, β-, and γ-CyDs, consisting of six, seven, and eight glucose units, respectively, are the most common natural CyDs. CyDs can improve the solubility, dissolution rate and bioavailability of the drugs, so the widespread use of CyDs is well known in the pharmaceutical field [47,48]. CyDs have been reported to interact with cell membrane constituents such as cholesterol and phospholipids, resulting in the induction of hemolysis of human and rabbit red blood cells (RRBC) [49–51]. Additionally, we have reported that CyDs induced hemolysis at high concentration: the magnitude of hemolytic activity of CyDs in human erythrocytes increased in the order of γ-CyD < α-CyD < 2-hydroxypropyl-β-CyD (HP-β-CyD) < β-CyD < 2,3,6-tri-*O*-methyl-β-CyD (TM-β-CyD) < 2,6-di-*O*-methyl-β-CyD (DM-β-CyD) [52]. The CyD-induced hemolysis is probably a secondary event resulting from the membrane disruption which elicited the removal of membrane components from erythrocytes [53]. The species and amounts of released components are dependent upon the cavity size of CyDs. The removal of cholesterol and proteins from the biomembranes is significant for β-CyD, which α-CyD releases phospholipids selectively. In addition, the hemolytic activity of methylated CyDs is well known to be rather high, compared to natural CyDs. Recently, we reported that DM-β-CyD and methyl-β-CyD (M-β-CyD) induced morphological changes in RRBC from discocyte to echinocyte through the extraction of cholesterol from cholesterol-rich lipid rafts [54], while 2,6-di-*O*-methyl-α-CyD (DM-α-CyD) induced morphological changes from discocyte to stomatocyte by the extraction of sphingomyelin from sphingolipid-rich lipid rafts, but not extraction of cholesterol [54]. Moreover, we demonstrated that DM-β-CyD induces apoptosis through the PI3K-Akt-Bad pathway, resulting from cholesterol depletion in cholesterol-rich lipid rafts, whereas DM-α-CyD induces necrosis, resulting from sphingolipids depletion in sphingolipid-rich lipid rafts [55]. Therefore, CyDs surely have the novel sensing function to release membrane components from biomembranes.

**Figure 2.**
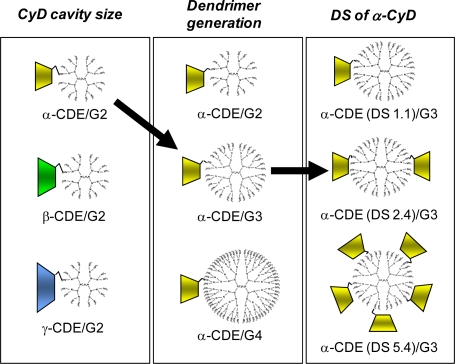
Optimization study of CDE as non-viral carriers.

Regarding the delivery of nucleic acid drugs using CyDs, it is acknowledged that CyDs interact with nucleic acids only very slightly [56]. Therefore, the potential of CyDs as carriers for nucleic acids on the basis of their direct interaction would not be expected. In view of this idea, the alternative use of CyDs for carriers of nucleic acids has been required. In addition, the use of CyD and its derivatives for increased transformation efficiency of competent bacterial cells through the interaction between CyDs and bacterial cell wall, not DNA [57]. Meanwhile, Davis and co-workers have reported a number of uses of β-CyD-containing polycations (CDP) with adamantine-PEG or adamantine-PEG-transferrin for gene, DNAzyme and siRNA transfer [46,58–61]. On the other hand, the widespread use of various CyD-appended polymers and polyrotaxanes as gene carriers has been reported, e.g., cationic star polymers consisting of α-CyD core and oligoethylenimine arms [62], PPI dendrimer graft β-CyD [63], low molecular weight PEI cross-linked by HP-β-CyD or HP-γ-CyD [64], low molecular weight PEIs linked by β-CyD [65], linear PEI through γ-CyD and biocleavable polyrotaxane [66], cationic supramolecules consisting of oligoethylenimine-grafted α-CyDs [67] and chitosan/CyD nanoparticles for the airway epithelium [68]. On the other hand, we have reported that CyD-conjugated dendrimers would have a significant impact as non-viral vectors (Figure 2), e.g., we prepared dendrimers (G2, G3, G4) conjugates (CDE) with CyDs [69–71]. Here the reasons why we used dendrimers with low generation and CyDs were their low cytotoxicity and endosome-disrupting effects through the release of membrane components from endosomal membranes after endocytosis, respectively. Of three CDE (G2) with α-, β- or γ-CyD at the molar ratio of 1:1 (dendrimer:CyD), dendrimers (G2) functionalized with α-CyD [α-CDE (G2)] showed luciferase gene expression about 100 times higher than for unfunctionalized PAMAM or for non-covalent mixtures of dendrimer and α-CyD, when pDNA encoding luciferase gene was used [69]. Of various α-CDEs, α-CDE (G3) with the degree of substitution (DS) of 2.4 [α-CDE (G3, DS2)] was revealed to have best transfection efficiency with low cytotoxicity, i.e., the gene transfer activity of α-CDE (G3, DS2) was found to be superior to commercially-available transfection reagents such as TransFast™ (TF) and Lipofectin™ (LF) [70,71]. Moreover, α-CDE (G3, DS2) was found to induce gene expression in spleen after intravenous injection of the pDNA complexes-containing suspension [71]. The enhanced gene transfer activity through the conjugation of α-CyD with dendrimer (G3) could be ascribed to the improved endosomal-escaping ability, i.e., the additive or synergetic effects of the proton sponge effects of dendrimers and the endosomal membrane-disrupting effects of α-CyD, based on the sensing function of α-CyD towards endosomal membranes [72]. However, the transfection efficiency of the pDNA complexes with α-CDEs seems to be still low, probably due to the lack of the translocation ability of the carriers into nucleus. Furthermore, we recently prepared dendrimer conjugates (G2) with glucuronylglucosyl-β-CyD [GUG-β-CDE (G2)] as a gene carrier and clarified the findings that gene transfer activity of GUG-β-CDE (G2) was superior to that of α-CDE (G2) and β-CDE (G2), with negligible cytotoxicity.

**Figure 3.**
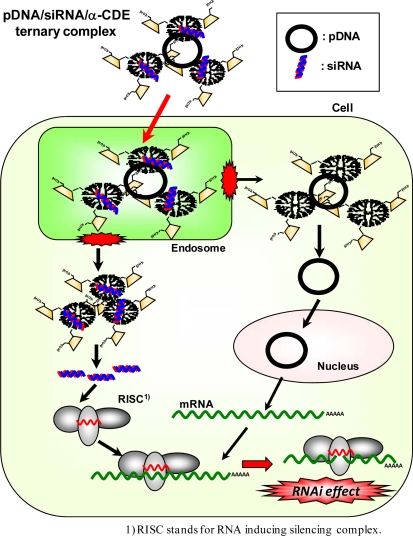
Proposed scheme of RNAi effects of the ternary complexes of pDNA/siRNA/α-CDE.

We have revealed that α-CDE (G3, DS2) have potential as siRNA carriers (Figure 3) [73,74]. The luciferase reporter gene system has been widely used to evaluate the efficiency of the siRNA carrier. Firstly, we used the cotransfection system: the ternary complex of luciferase reporter plasmids with siRNA duplexes and a carrier is transfected and it is acknowledged to be useful for simple evaluation of the RNAi effect at the early phase [73]. Then, we examined the sequence specific gene silencing effects using α-CDE (G3, DS2) as a siRNA carrier. Here we evaluated by measuring of luciferase activity after transfected with ternary complexes of DNA/siRNA/α-CDE (G3, DS2) and compared its RNAi effect with the other commercial transfection reagents, i.e. Lipofectamine™ 2000 (L2000), TF and LF. The ternary complex of α-CDE (G3, DS2) induced sequence-specific gene silencing without the off-target effect and its luciferase activity was reduced to half of a control. Meanwhile, all of the commercial transfection reagents used also displayed pGL2 siRNA specific inhibition, but L2000 and TF had non-specific effects on pGL3 siRNA and gave the unstable gene expression effect, compared with α-CDE (G3, DS2). Secondly, we used the transfection system: the binary complex of siRNA/α-CDE (G3, DS2) was transfected to cells transiently and stably expressing luciferase reporter genes. In these systems, α-CDE (G3, DS2) was found to have the potent RNAi effects, compared to L2000 and TF [73]. Thus, α-CDE (G3, DS2) may be a new candidate for a potential therapeutic agent for a siRNA carrier. Thirdly, we examined the *in vivo* RNAi effect in mice inoculated Colon-26 tumor cells stably expressing luciferase reporter gene [75]. When siRNA complex with α-CDE (G3, DS2) was intratumorally injected, luciferase activity was significantly decreased, but siRNA complexes with L2000 provided the off-target effects. Thus, α-CDE (G3, DS2) has the potential as a siRNA carrier *in vitro* and *in vivo*.

Recently, shRNA has been developed in order to improve duration of the RNAi effect [8]. Therefore, the shRNA transfer activity of α-CDE (G3, DS2) was compared with that of dendrimer (G3). α-CDE (G3, DS2) formed a stable and condensed complex with shRNA and induced a conformational transition of shRNA in solution even in the low charge ratios. In addition, α-CDE (G3, DS2) markedly inhibited the enzymatic degradation of shRNA by DNase I. The shRNA complex with α-CDE (G3, DS2) at the charge ratio of 20/1 (carrier/shRNA) elicited the most potent RNAi effects in cells transiently and stably expressing the GL3 and GL2 luciferase genes without cytotoxicity. Besides, the RNAi effects were strikingly enhanced by the further addition of the adequate amounts of siRNA to the shRNA complex with α-CDE (G3, DS2). Taken together, the prominent RNAi effects of the shRNA complex with α-CDE could be attributed to its stabilizing effect on enzymatic degradation of shRNA and negligible cytotoxicity. These results suggest that α-CDE (G3, DS2) has the potential to be a novel carrier for shRNA as well as siRNA.

## 4. Sugar-appended α-CDEs as DNA Carriers

**Figure 4.**
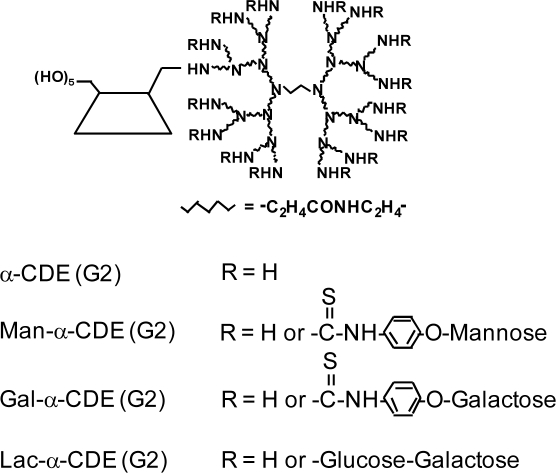
Chemical structures of α-CDE (G2) and sugar-appended α-CDEs (G2).

α-CDE (G3, DS2) possesses the potential to be a novel carrier for nucleic acid drugs, but the lack of cell-specific gene transfer activity of α-CDEs has been shown. A carrier system needs to fulfill the following requirements to be a promising candidate for *in vivo* gene delivery. The carrier should be able to efficiently accumulate in specific target tissues with the lack of toxicity and immunogenicity, and deliver the intact gene into the nucleus of target cell to get high levels of gene expression. Instead of viral vectors, synthetic carriers such as polymers have become an attractive alternative due to their relative safety and their lack of restraints on the size of the pDNA to be delivered. Among the non-viral methods, the glycofection method has recently come to attention [76]. Glycosylated polymers are used for transfection and interact with pDNA to give a glycoplex [77]. In general, glycoplexes are used for delivery to the specific cells and/or to augment gene transfer activity [78]. For example, a mannosylated PEI has high transfection efficiency to macrophages and dendritic cells, which were mediated by the mannose receptor and DEC-205, respectively [79]. Additionally, galactosylated PEI has high transfection efficiency to hepatocytes expressing an asialoglycoprotein receptor (AgpR) [80]. Furthermore, some findings showing glycosyl residues to be very promising candidates of a nuclear targeting signal have been reported [78]. Thus, glycosylation of polymers is an effective method to deliver gene to target cells and/or to enhance gene transfer activity. To possess the cell-specific gene transfer activity of α-CDE (G3, DS2), we prepared the three types of sugar-appended α-CDEs: mannosylated α-CDEs [Man-α-CDEs (G2, G3)] [67,81], galactosylated α-CDEs [Gal-α-CDEs (G2)] [82] and lactosylated α-CDEs [Lac-α-CDE (G2)] [75] with the various degree of substitution (DS) of these sugar moieties (Figure 4).

Firstly, to achieve antigen presenting cells (APC)-specific gene delivery of α-CDE (G2), we prepared Man-α-CDE (G2) with the various DS of the mannose moiety (DSM) and evaluated their gene transfer activity in a variety of cells [83], because APC express mannose receptors. Man-α-CDEs (G2, DSM3, 5) were found to have much higher gene transfer activity than dendrimer, α-CDE (G2) and Man-α-CDE (G2, DSM1, 8) in various cells, which are independent of the expression of cell surface mannose receptors. The surface plasmon resonance (SPR) study demonstrated that the specific binding activity of Man-α-CDE (G2, DSM3) to concanavalin A, a mannose lectin, was not very strong. It should be noted that Man-α-CDE (G2, DSM3) provided gene transfer activity higher than dendrimer and α-CDE (G2) in kidney 12 h after intravenous injection in mice. These results suggest the potential use of Man-α-CDE (G2, DSM3) as a non-viral vector, although Man-α-CDE (G2, DSM3) did not show cell-specific gene delivery.

Secondly, to improve APC-specific gene transfer activity of Man-α-CDE (G2), we prepared Man-α-CDEs (G3) with various DSM (5, 10, 13, 20) and compared their cytotoxicity and gene transfer activity, and elucidated the enhancing mechanism for the activity [81]. Of the various carriers used here, Man-α-CDE (G3, DSM10) provided the highest gene transfer activity in NR8383, A549, NIH3T3 and HepG2 cells and the activity of Man-α-CDE (G3, DSM10) was not decreased by the addition of 10% serum in A549 cells. Additionally, no cytotoxicity of the polyplex with Man-α-CDE (G3, DSM10) was observed in A549 and NIH3T3 cells up to the charge ratio of 200:1 (carrier:pDNA). However, the gene transfer activity of Man-α-CDE (G3, DSM10) was independent of the expression of mannose receptors. Interestingly, Alexa-pDNA complex with TRITC-Man-α-CDE (G3, DSM10), but not the complex with TRITC-α-CDE (G3), was found to translocate to the nucleus at 24 h after incubation in A549 cells. HVJ-E vector including mannan, but neither the vector alone nor the vector including dextran, suppressed the nuclear localization of TRITC-Man-α-CDE (G3, DSM10) to a striking degree after 24 h incubation in A549 cells. These results suggest that Man-α-CDE (G3, DSM10) has less cytotoxicity and prominent gene transfer activity through not only its serum resistant and endosome-escaping abilities but also nuclear localization ability, although Man-α-CDE (G3, DSM10) did not elicit cell-specific gene delivery.

**Figure 5.**
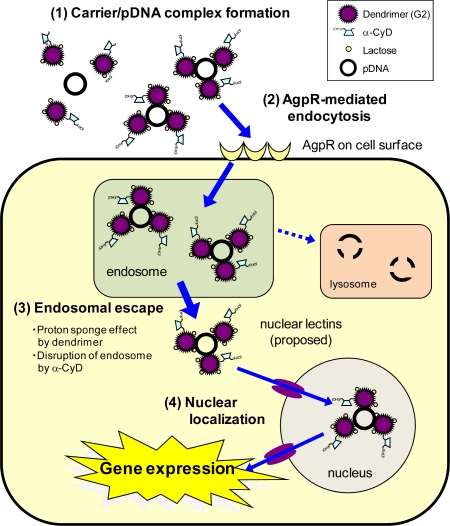
Proposed scheme for improved effects of gene transfer activity by Lac-α-CDE (G2, DSL3).

Thirdly, to improve gene transfer efficiency and/or achieve cell-specific gene delivery of α-CDE (G2), we prepared α-CDE bearing galactose [Gal-α-CDE (G2)] with the various DS of the galactose moiety (DSG) as a novel non-viral vector [82]. Gal-α-CDE (G2, DSG4) was found to have much higher gene transfer activity than dendrimer, α-CDE (G2) and Gal-α-CDEs (G2, DSG8, 15) in HepG2, NIH3T3 and A549 cells, which are independent of AgpR expression. Gene transfer activity of Gal-α-CDE (G2, DSG4) was insensitive to the existence of competitors (asialofetuin and galactose) and serum. These results suggest the potential use of Gal-α-CDE (G2, DSG4) as a non-viral vector in various cells, although Gal-α-CDE (G2, DSG4) did not have a cell-specific gene transfer activity. Here we envisaged that α-D-mannopyranosylphenyl isothiocyanate and α-D-galactopyranosylphenyl isothio-cyanate as a space between sugar moiety and dendrimer in the Man-α-CDEs (G2, G3) and Gal-α-CDE (G2) may be involved in the lack of cell-specific gene delivery of these sugar-appended α-CDEs owing to the short length of the spacer. Thereby, we prepared, therefore, α-CDEs bearing α-lactose, a disaccharide formed from α-glucose and α-galactose (Lac-α-CDE) without using the spacer [75]. Of Lac-α-CDEs (G2) having various DS of lactose moiety (DLS1 ,3, 5, 6, 10), Lac-α-CDE (G2, DSL3) was found to have the highest gene transfer activity than dendrimer, α-CDE and the other Lac-α-CDEs in HepG2 cells, AgpR-positive cells, but not in A549 cells, AgpR-negative cells. In addition, the luciferase gene transfer activity of Lac-α-CDE (G2, DSL3) was markedly suppressed in HepG2 cells by adding asialofetuin, a competitor against AgpR, but not bovine serum albumin (BSA). Furthermore, the flow cytometric study showed that cellular association of polyplex with Lac-α-CDE (G2, DSL3) was also suppressed by addition of asialofetuin, not BSA, in HepG2 cells. Thus, it should be noted that Lac-α-CDE (G2, DSL3) provided hepatocyte-selective gene transfer activity through the binding of the carrier to AgpR on HepG2 cells. In fact, the SPR study clearly demonstrated that the association constant of Lac-α-CDE (G2, DSL3) to peanut lectin, a galactose lectin, was approximately 100-fold higher than that of α-CDE (G2). Moreover, Alexa-pDNA complex with TRITC-Lac-α-CDE (G2, DSL3), but not the complex with TRITC-α-CDE (G2), was found to translocate to nucleus in HepG2 cells, suggesting the lactose-mediated nuclear translocation. These proposed hepatocyte-selective gene transfer behaviors of Lac-α-CDE (G2, DSL3) are shown in Figure 5. Importantly, the AgpR-dependent gene delivery of Lac-α-CDE (G2, DSL3) was observed *in vivo*: Lac-α-CDE (G2, DSL3) provided gene transfer activity much higher than α-CDE (G2) in parenchymal cells and much lower than in spleen 12 h after intravenous injection in mice. In addition, neither cytotoxicity nor change in serum chemistry value was observed, when the complex of pDNA with Lac-α-CDE (G2, DSL3) was applied to culture cells and mice, respectively. Hence, these results hold promise for the potential use of Lac-α-CDE (G2, DSL3) as a hepatocyte-selective non-viral vector with negligible cytotoxicity.

## 5. Folate-appended α-CDEs as DNA Carriers

**Figure 6.**
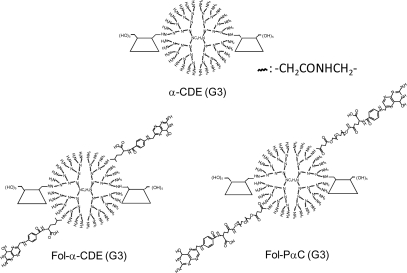
Chemical structures of α-CDE (G3), Fol-α-CDE (G3) and Fol-PαC (G3).

Targeting of the folate receptor (FR) had received much attention in recent years, since the FR has been shown to be over expressed in human cancer cells [84]. Additionally, folic acid is a relatively small molecule (MW 441 Da) which consequently has only limited effects on the dimensions of the carrier system. Some papers regarding folate-appended dendrimers have been published so far. For example, Konda *et al*. reported the folate-dendrimer MRI contrast agents to the high affinity folate receptor expressed in ovarian tumor xenografts [85]. Shukla *et al*. demonstrated that folate receptor-targeted boronated PAMAM dendrimers are potential agents for neutron capture therapy [86]. In addition, Singh *et al*. reported that folate-PEG-dendrimer conjugate was significantly safe and effective in tumor targeting for 5-fluorouracil, an anticancer drug, compared to a non-PEGylated formulation [87]. In an attempt to develop FR-overexpressing cancer cell-specific gene transfer carriers, we prepared folate-appended α-CDEs [Fol-α-CDE (G3)] and folate-PEG-appended α-CDEs [Fol-PαC (G3)] (Figure 6) and evaluated their potential as a novel cell-specific gene transfer carrier. Gene transfer activity of Fol-α-CDEs (DS of folate; DSF2, 5, 7) was lower than that of α-CDE (G3) in KB cells, FR-overexpressing cells. Of the three Fol-PαC (G3, DSF2, 5, 7), Fol-PαC (G3, DSF5) had the highest gene transfer activity in KB cells. This activity was significantly higher than that of α-CDE (G3) in KB cells, but not in A549 cells, FR-negative cells. The cellular uptake of the pDNA complexes with Fol-PαC (G3, DSF5) was inhibited by adding folic acid as a competitor of FR, suggesting the FR-mediated endocytosis. In fact, the SPR data indicated that the association constant of Fol-PαC (G3, DSF5) with folate binding protein (FBP) was approximately 320-fold higher than that of α-CDE (G3). No cytotoxicity of the DNA complex with Fol-PαC (G3, DSF5) was observed in KB cells or A549 cells up to the charge ratio of 100:1 (carrier:DNA), although the DNA complexes with PEI (10 kDa, 25 kDa) showed cytotoxicity even at a charge ratio of 10:1 (carrier:DNA). Most recently, we revealed that DNA complex with Fol-PαC (G3, DSF5) elicited *in-vivo* gene transfer activity in tumor tissues in mice. In conclusion, potentially, Fol-PαC (G3, DSF5) could be used as a FR-overexpressing cancer cell-selective gene transfer carrier because of FR-mediated gene delivery and the extremely low cytotoxicity.

## 6. Conclusions

Many attempts have been made to design and evaluate CyD conjugates with polymers for DNA, shRNA and siRNA carriers. In this review, we have demonstrated the potential of α-CDEs as DNA, shRNA and siRNA carriers. However, their clinical use may be still very limited, so we have sought to extend the function of α-CDEs. Moreover, the development of the sustained release systems of polyplexes with α-CDEs would be required. Elaborate studies are further required to develop novel carriers for various nucleic acid drugs such as gene, shRNA, siRNA, decoy DNA, antisense DNA, ribozyme and aptamers. The future should see certain clinical use products using CyD-containing carriers for DNA and RNA.
